# Regulation of muscle springiness and hardness: the role of *myo*-inositol in enhancing fish flesh texture

**DOI:** 10.1016/j.fochx.2025.102788

**Published:** 2025-07-12

**Authors:** Meiqi Wang, Lin Feng, Pei Wu, Yang Liu, Hongmei Ren, Xiaowan Jin, Xiaoqiu Zhou, Weidan Jiang

**Affiliations:** a Animal Nutrition Institute, Sichuan Agricultural University, Chengdu 611130, China; b Fish Nutrition and Safety Production University Key Laboratory of Sichuan Province, Sichuan Agricultural University, Chengdu 611130, China; c Key Laboratory of Animal Disease-Resistance Nutrition, Ministry of Education, Ministry of Agriculture and Rural Affairs, Key Laboratory of Sichuan Province, Sichuan 611130, China

**Keywords:** Springiness, Hardness, Texture, Flavor, *Myo*-inositol, Flesh quality

## Abstract

As consumer demand for high-quality aquatic products continues to rise, enhancing the flesh texture of these products, including attributes such as hardness and springiness, has become increasingly critical. To examine the beneficial impacts of *myo*-inositol (MI) on the flesh quality of grass carp (*Ctenopharyngodon idella*), six different diets containing 35 (basal diet), 98, 195, 292, 389, and 487 mg/kg of MI were used for an 8-week period. Our results demonstrate that MI supplementation (292–389 mg/kg) enhances both the springiness and hardness of fish. The increase in springiness and hardness is likely attributable to MI's promotion of elastin and collagen synthesis while simultaneously inhibiting their degradation. Furthermore, 195–487 mg/kg MI enhances fish freshness and flavor by mitigating postmortem ATP degradation and modulating the free amino acid profile. This study establishes a foundation for investigating the contribution of MI to enhancing the flesh texture of adult grass carp.

## Highlights

• *Myo*-inositol enhances muscle tissue springiness and hardness, thereby contributing to an improved flesh texture.
• *Myo*-inositol reduced the decomposition of ATP in muscle after slaughter, slowed down the decrease of pH, and improved the freshness of fish.
• *Myo*-inositol improved the flavor of fish.

## 1 Introduction

Fish serves as a high-quality protein source in the human diet. As living standards have improved, consumer demand for higher-quality fish has increased correspondingly. Texture, as a sensory property encompassing characteristics such as hardness, springiness, chewiness, and adhesiveness, is widely regarded as the paramount factor influencing consumer satisfaction and perception of flesh quality. Typically, high-quality fish is considered firm and cohesive in texture (Matarneh et al., 2021). Dietary intervention is a commonly employed strategy for manipulating flesh quality. Vitamins are organic compounds essential for the normal growth, development, and function of the animal organisms (Wang et al., 2024). *Myo*-inositol (MI), a vitamin-like nutrient, serves as a critical precursor for phosphatidylinositol synthesis, playing an essential role in the structural organization of cell membranes and transmembrane signal transduction. MI exhibits unique functions not commonly observed in other nutrients. For example, while vitamins C and E primarily enhance fish flesh texture via their antioxidant properties, MI may directly regulate the transcription of genes associated with texture. Furthermore, given the limited capacity of fish to synthesize MI endogenously, exogenous MI supplementation is crucial for promoting growth and maintaining health in fish (Shi et al., 2021). Previous studies have demonstrated that MI can enhance the flesh quality of Chinese mitten crabs (*Eriocheir sinensis*) (Bu et al., 2022). As of now, no existing studies have documented the effects of MI on flesh texture.

Among the texture properties, springiness is a critical parameter for evaluating the freshness of fish flesh. It quantifies the extent of recovery between the first and second compressions during mastication. The parameter is significantly influenced by the structure of the connective tissue, with elastin being the primary contributor, comprising 0.6 % to 7.9 % of the connective tissue (Diller & Tabor, 2022). The expression and formation of proelastin are regulated by numerous effector factors. Numerous studies have demonstrated that the transforming growth factor β-1 (TGF-β1)/Smads signaling pathway promotes elastin formation (Katsuta et al., 2008). Additionally, TGF-β1 suppresses the breakdown of elastin by decreasing the concentrations and enzymatic activity of elastin-degrading proteases, such as matrix metalloproteinases (MMP-2 and MMP-9) (Dai et al., 2005). Prior research has shown that MI inhibits the expression levels of TGF-β1 and reduces the secretion of MMP-9 in breast cells under inflammatory conditions (Monti et al., 2023). Furthermore, the EGF/MEK/ERK signaling cascade was antagonized by TGF-β1 signaling, thereby mitigating the positive influence of TGF-β1 on proelastin mRNA stability (DiCamillo et al., 2006). While research has indicated a connection between MI and the regulation of effector factors involved in elastin synthesis and degradation, the specific mechanism by which MI regulates elastin formation in fish and its impact on springiness remains unclear and warrants further investigation.

Muscle hardness is a critical determinant of fish flesh quality, closely associated with the quantity, distribution, and degree of crosslinking of collagen (Wang et al., 2024). As of now, there have been no studies reported on the impact of MI on collagen biosynthesis in fish flesh. Similar to livestock, the predominant form of collagen in fish flesh is Type I collagen (Liang et al., 2021). Collagen genes undergo transcription and translation to synthesize procollagen, which subsequently forms mature fibers through covalent cross-linking (Gelse et al., 2003). Collagen biosynthesis is mediated by a variety of biological pathways, whereas TGF-β1 and La ribonucleoprotein structural domain family member 6 (LARP6) are thought to be central to transcription and translation, respectively (Wen et al., 2023). As a precursor to second messengers, MI modulates various signal transduction pathways and metabolic circuits within cells. It has been reported that MI has insulin-like activity, which may be due to the role of inositol phosphoglycans (IPG) as the second messenger of insulin (Croze & Soulage, 2013). In a mice study, insulin-like growth factor-1 (IGF-1) was found to enhance the expression of LARP6 in the aorta, thereby upregulating the capacity for Type I collagen synthesis (Blackstock et al., 2014). Therefore, we hypothesize that MI may affect collagen biosynthesis thereby affecting flesh texture deserves further investigation.

Texture serves as an important indicator of freshness, as it tends to become softer in the post-slaughter state of fish. Immediately after death, autolysis reactions controlled by natural enzymes occur in fish tissues. Among these reactions, the degradation of ATP predominates [ATP → adenosine monophosphate (AMP) → inosine monophosphate (IMP) → inosine (HxR) → hypoxanthine (Hx)] (Hong et al., 2017), and the K value has been extensively utilized to assess fish freshness. A higher K value indicates greater ATP breakdown and lower freshness. Generally, a K value <20 % is considered the ideal freshness for consumption, whereas a K value of 60 % indicates the threshold of acceptable edibility (Cheng et al., 2015). However, the impact of MI on flesh freshness has not been documented. It has been reported that MI derivative inositol pyrophosphate regulates ATP concentration in mutant yeast (*Saccharomyces cerevisiae)* cells by regulating glycolysis/mitochondrial ratio (Szijgyarto et al., 2011). Therefore, we hypothesize that dietary MI may affect the freshness and texture in fish flesh after death, which needs further study.

This study systematically examined the impact of incorporating MI into feed on the post-mortem muscle quality of grass carp, with a focus on analyzing the dynamic changes in freshness indicators (e.g., pH value, ATP degradation, and K value) and flavor-related free amino acids. Additionally, this study elucidated the molecular mechanism by which MI regulates the synthesis and degradation of elastin and collagen, thereby influencing muscle elasticity and hardness. These findings provide novel theoretical insights for enhancing the post-mortem texture characteristics of aquatic animals. Furthermore, from a nutritional regulation perspective, this study establishes a scientific basis for refining aquaculture strategies and improving the edible quality of fish flesh.

## 2 Materials and methods

### 2.1 Experimental diets

The composition and nutritional content of basal diets are detailed in Table S1. Six levels of MI premix were reached as a supplement to a basal diet: Basal diet (without supplement), 100, 200, 300, 400, and 500 mg MI/kg diets. All diets were blended evenly, dried in an oven, and then refrigerated at 4 °C after being ground into granules. To assess MI levels in the six experimental diets, high-performance liquid chromatography (HPLC) was utilized, following the protocol outlined by Frieler (Frieler et al., 2009), 35 (basal diet), 98, 195, 292, 389 and 487 mg/kg, respectively. AOAC standards were used to determine the crude lipid and crude protein contents of samples in dry matter (AOAC, 2005).

### 2.2 Fish and experimental design

There were 450 healthy grass carps kept in barrels with a diameter of 1 m and a height of 1 m. After purchase, acclimate the environment for four weeks. In the pre-feeding period, adult grass carps (704.84 ± 0.91 g) were randomly distributed among 6 groups with three replications each of 25 fish. A basal diet without supplementation of MI was fed to six groups of grass carp for 14 days (Li et al., 2017a). For 8 weeks, grass carp were fed 4 meals (07:00, 11:00, 15:00, and 19.00) every day, and no grass carp perished (Wen et al., 2014). Throughout the trial, daily monitoring was conducted for temperature, dissolved oxygen concentrations, and pH levels. A temperature range of 26.0 to 30.7 °C, oxygen concentrations of >6.0 mg/L and pH levels of 7.0 to 7.5 were determined. The impact of dietary MI on growth performance is detailed in Table S2.

### 2.3 Samples collection and analysis

A 24-h fast was conducted following the conclusion of the experiment on all fish. Subsequently, several growth performance measures were determined by weighing the fish in each experimental breeding barrel. Post-weighing, we selected 12 fish at random from each treatment group and anesthetized them with benzocaine bath (50 mg/L) and euthanized them with a cranial blow. The euthanasia protocol adhered to American Veterinary Medical Association (AVMA) guidelines. The left back muscle sample (6 × 6 cm) of the fish was quickly taken and divided into three parts, which were used to determine pH value, shear force and cooking loss, respectively. In each group, three grass carps were randomly selected for muscle collection, which was then fixed in 3 % glutaraldehyde and 4 % paraformaldehyde solutions, respectively, until electron micrography and histologically analysis.

The muscle samples were continuously maintained on ice throughout the process, and pH measurements at 0 h (pH_0h_) and 24 h (pH_24h_) were taken using a calibrated pH probe (Testo 205 pH meter, Testo AG Company, Lenzkirch, Germany). In order to measure cooking loss, muscle samples underwent heating at 70 °C for a period of 20 min, and their weights were recorded before and after heating.

As instructed, a microplate method was used to measure muscle lactate content (kit No. A019–2-1). Collagen content in muscle samples was determined using a hydroxyproline (HYP) kit, with the collagen content calculated by multiplying the HYP concentration by a factor of 8 (kit No. A030–2-1). Colorimetry was used to measure ATP content in muscle (kit No. A095–1-1). The contents of malondialdehyde (MDA, kit NO. A003–1-1), protein carbonyl (PC, kit NO. A087–1-1) and enzyme activity of superoxide dismutase (T-SOD, kit NO. A001–3-1) and catalase (CAT, kit NO. A007–1-1) in muscle were measured using respective kits (Nanjing Jiancheng Institute of Bioengineering, China). The contents of adenosine monophosphate (AMP, kit No. YJ23669), inosine monophosphate (IMP, kit No. YJ56662), and inosine (HxR, kit No. YJ87551) in muscle were measured using respective kits (Mlbio, Shanghai, China) according to the manufacturer's instructions.

### 2.4 Free amino acid (FAA) analysis

Fully automatic amino acid analyzers (Biochrom, UK) were utilized to examine the composition of free amino acids in muscle samples. To prepare the sample homogenate, first collect 400 mL of the homogenate and add 800 μL of a 0.1 g/mL salicylic acid standard solution. Allow the mixture to stand for 30 min at 25–27 °C. Subsequently, centrifuge at 4 °C for 5 min and then filter to acquire the filtrate for analysis.

### 2.5 Freshness and texture analysis

The freshness analyzer was employed to measure the K value of fish flesh. Fish flesh was initially cut into 5 × 5 mm sections using scissors, mixed with 600 μL of extract A, then further minced and homogenized with extract B. The sample solution pH was adjusted to 5–8 prior to analysis with the MFT2 instrument (ENSOUL TECHNOLOGY LTD, Beijing, China).

For texture analysis, cooked muscle samples were sectioned into 1.0 cm × 1.0 cm × 0.5 cm blocks. Texture profile analysis (TPA) parameters (hardness, cohesion, adhesion, elasticity, chewiness) were quantified using a Brookfield texture analyzer (USA). Shear force measurements were performed perpendicularly to muscle under standardized conditions: P50 probe (50 mm diameter, 75 % compression ratio, 0.1 N trigger force, 5 s interval), with automated pre-test (1.0 mm/s), test (5.0 mm/s), and post-test (5.0 mm/s) speed (Wang et al., 2025).

### 2.6 Electronic tongue

The flavors of fresh and cooked fish were examined using an electronic tongue (Alpha M.O.S., Astree). After accurately weighing a 2.00 g piece of tissue, it was homogenized in 30 ml of high-purity water and centrifuged at 10,000*g* for 10 min at 4 °C after a 15-min settling time. After filtering and diluting the supernatant with high purity water to 100 ml, the samples were analyzed using an e-tongue.

### 2.7 Histological observation

After sampling, a GD fixation solution (G1111, Servicebio, China) was used to preserve the muscle tissue as well as embed it in paraffin. In the next step, 5 μm sections of the tissue were cut, dewaxed with xylene, and sirius red was used to stain the collagen fibers within the muscle tissue. The Verhoeff-Van Gieson (EVG) staining method can separately color the elastic fibers and collagen fibers in tissues. The elastic fibers are stained black, while the collagen fibers are stained red. We used Image J software to evaluate the collagen region. The image is initially converted into an 8-bit grayscale format and subsequently processed using the automatic thresholding method. This process is further refined by integrating the Default mode with manual adjustments to ensure comprehensive coverage of the collagen regions. Concurrently, the binarization outcome is evaluated via the real-time preview feature to confirm the precision of the threshold selection.

### 2.8 Electron microscope analysis

We postfix samples with 1 % osmium tetroxide after prefixing them with 3 % glutaraldehyde. After 3 h of immersion in 1 % tannic acid, the samples were washed with distilled water for several hours before being fixed in 1 % osmium tetroxide-0.1 M phosphate buffer for 1 h and buried with liquid paraffin. Once cut, the specimens were stained with eosin and freeze-dried. Once dried and coated with gold, the samples were examined.

### 2.9 Quantitative real-time PCR analysis

Assays were performed using Trizol (Takara, China) to prepare total RNA extracts. The cDNA was synthesized using a cDNA synthesis kit (Vazyme, China). The Quant-Studio 5 Flex system from Applied Biosystems, CA, USA, was used to conduct the RT-PCR, with a final reaction volume of 10 μL, employing the fast SYBR green qPCR master xix kit (Aidlab, China). Gene expression quantification was performed using the 2^-ΔΔCT^ method, with β-actin acting as the internal standard. In Table S3, we provide the primer sequences.

### 2.10 Western blotting

We performed radio immunoprecipitation assays (RIPA) (Beyotime, China) on muscle samples to extract total protein. Using the kit, we extracted nuclear proteins (No. P0027. Beyotime, China). Each sample was tested for protein concentration using protein detection kits of BCA (Nanjing Jiancheng Bioengineering Institute, China). Following SDS-PAGE gel separation, the protein samples were transferred to PVDF membranes, and overnight incubation with primary antibodies was carried out at 4 °C. Incubation of the primary antibody was followed by addition of the secondary antibody to the PVDF membrane. With an enhanced chemiluminescence system (ECL), the bands were visualized, and Image J was utilized for analyzing the results. Table S4 provides information about antibodies.

### 2.11 Statistical analysis

This experiment follows a completely random design. Using SPSS version 27.0, a one-way ANOVA was carried out, and Tukey's multiple range test was applied (SPSS Inc., Chicago, IL, USA). Pearson correlation analysis was employed to evaluate the linear relationships between variables. Statistical significance was established when the *P* < 0.05. Data are presented as the mean ± SEM. Images were generated using Origin 2021 and GraphPad Prism 8 software.

## 3 Results

### 3.1 Effect of myo-inositol on free amino acid composition in muscle

**Fig. 1:**
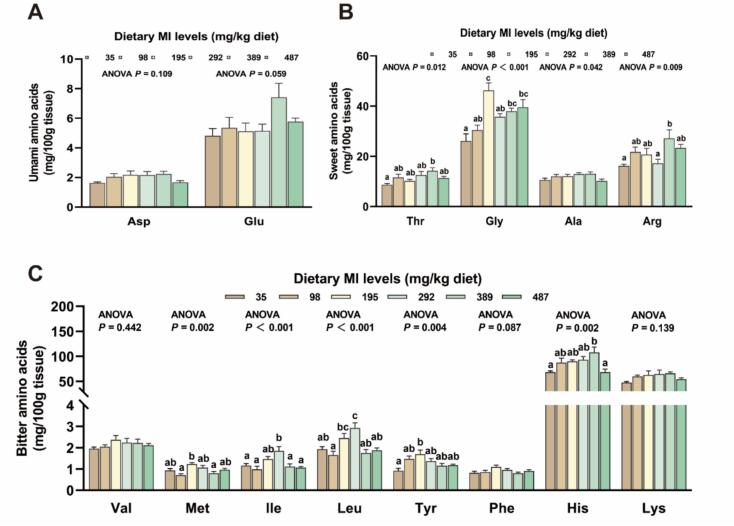
The effect of *myo*-inositol on free amino acid composition in the muscle of grass carp (mg/100 g tissue). Values are mean ± SEM. *n* = 6. *P* < 0.05 was considered as significant difference. Glu: Glutamic acid; Asp: Aspartic acid; Thr: Threonine; Gly: Glycine; Ala: Alanine; Phe: Phenylalanine; Val: Valine; Ser: Serine; Met: Methionine; Ile: Isoleucine; Leu: leucine; Tyr: Tyrosine; Lys: Lysine; His: Histidine; Arg: Arginine.

FAA composition is the main factor affecting flavor. The influence of MI on FAA composition is detailed in Fig. 1. Compared with the MI deficiency group (35 mg MI/kg), the content of umami FAA glutamic acid (Glu), sweetness FAA threonine (Thr), glycine (Gly) and alanine (Ala), savoriness FAA serine (Ser) and arginine (Arg) in the MI addition amount of 389 mg/kg group was significantly enhanced (*P* < 0.05). Compared with the MI deficiency group, the contents of umami FAA lysine (Lys) and histidine (His) in the MI addition amount of 292 to 389 mg/kg group were significantly enhanced compared with the MI deficiency group (*P* < 0.05). The umami FAA aspartic acid (Asp) and valine (Val) in MI treatment groups did not differ significantly from each other (*P* > 0.05).

### 3.2 Key genes involved in muscle nutrition and texture regulation

Compared with the MI deficiency group (35 mg MI/kg), the 195–389 mg MI/kg group exhibited a significant enhanced in calpastatin (*CAST*), lipoprotein lipase (*LPL*), malate dehydrogenase (*MDH*), peroxisome proliferator-activated receptor gamma (*PPARγ*) and peroxisome proliferator-activated receptor gamma co-activator 1 alpha (*PGC-1α*) mRNA levels (*P* < 0.05). The correlation analysis revealed that the expression level of the CAST gene was significantly positively correlated with both the crude protein content in muscle nutritional components and the hardness parameter of texture. Furthermore, the expression levels of the *LPL*, *MDH*, *PPARγ*, and *PGC-1α* genes were also found to be significantly positively correlated with the crude lipid content in muscle tissue.

### 3.3 Texture properties of muscle

**Fig. 2:**
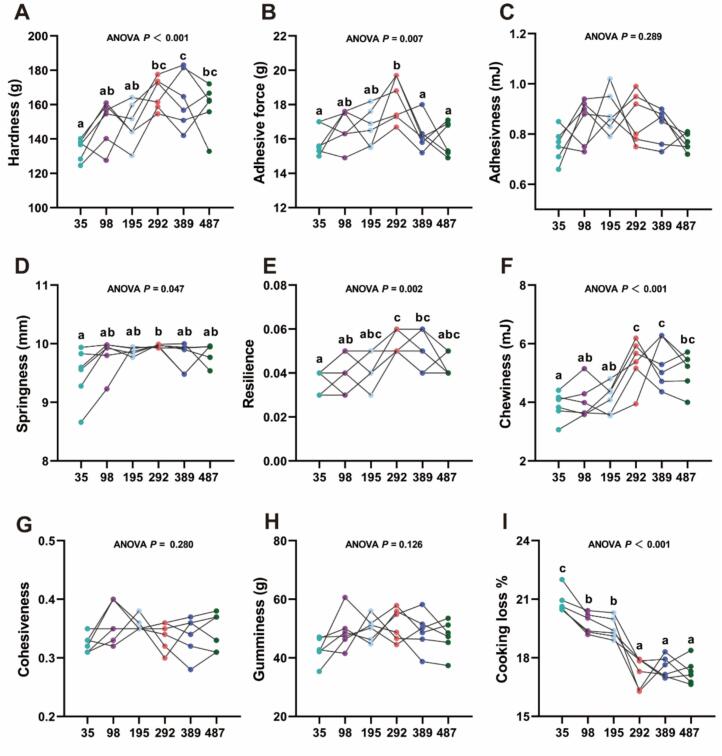
The effect of *myo*-inositol (MI) on texture parameters of grass carp muscle tissue. ^a-c^ mean values within the same row with different superscripts are significantly different (*P* < 0.05). Values are mean ± SEM (n = 6).

As illustrated in Fig. 2, compared to the MI-deficient group, the fish in the 292 mg MI/kg groups had significantly higher hardness, springiness, adhesive force, chewiness and resilience (*P* < 0.05). The cooking loss of 98 to 487 mg MI/kg group exhibited a significantly lower value compared to the deficiency group (*P* < 0.05). However, no significant differences in adhesiveness, gumminess and cohesiveness were noted between the groups (*P* > 0.05).

### 3.4 Effect of myo-inositol on muscle freshness and antioxidation in grass carp

**Fig. 3:**
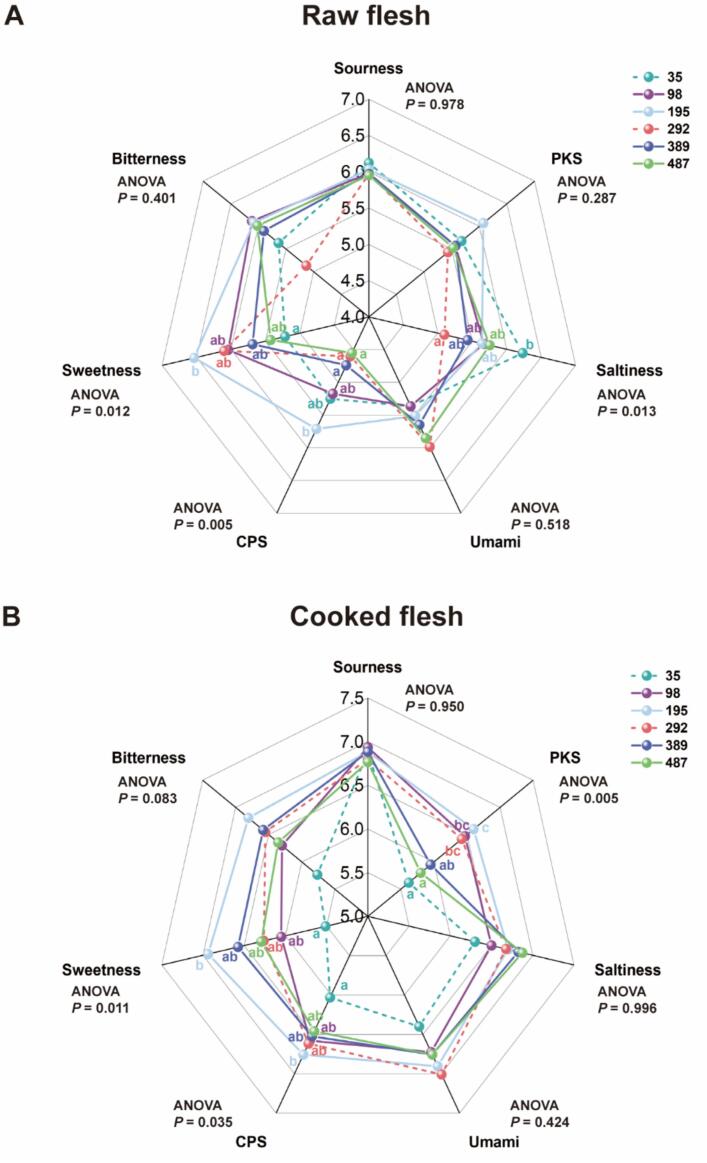

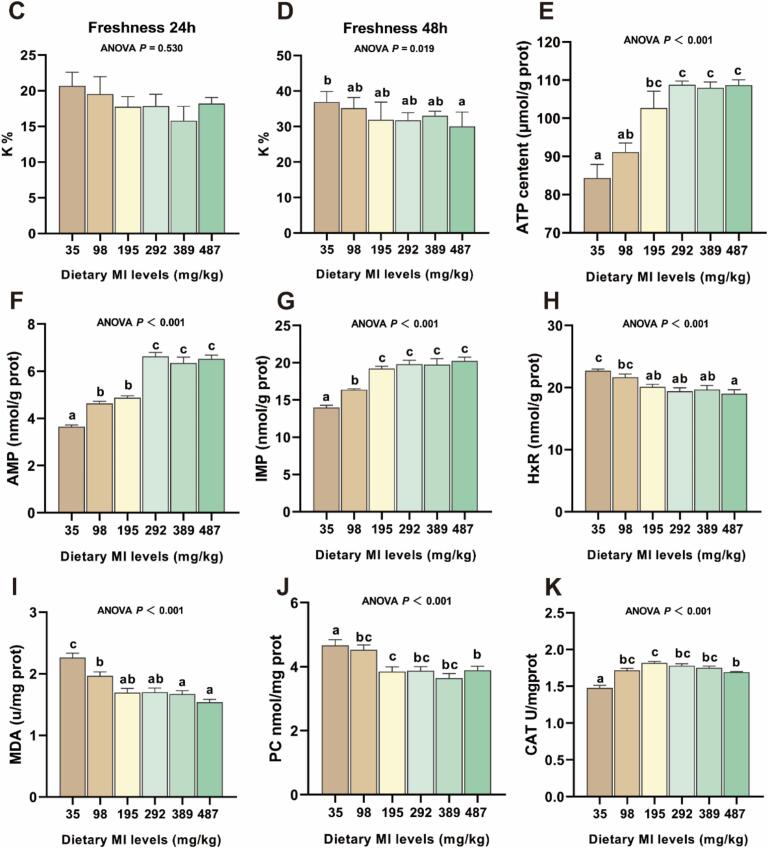

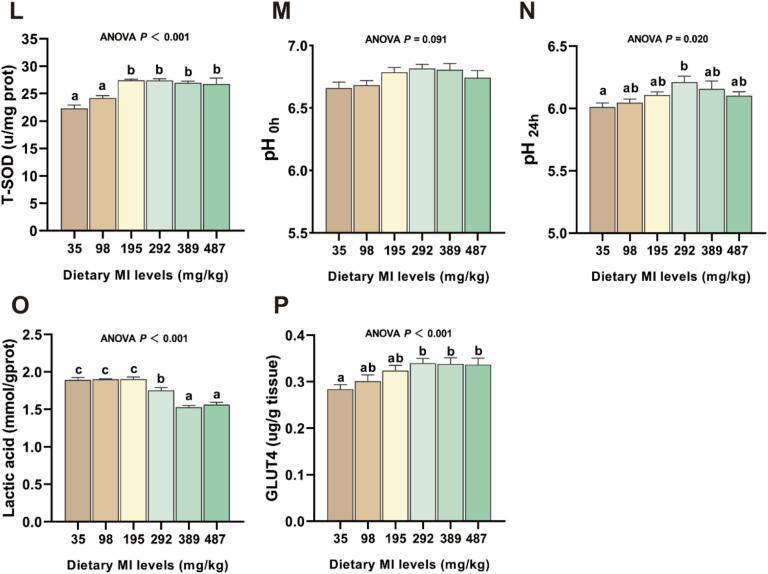
The effects of *myo*-inositol (MI) on the freshness and sensory characteristics of grass carp muscle tissue. (A) The raw flesh radar images detected from electronic tongue (n = 6); (B) The cooked flesh radar images detected from electronic tongue (n = 6). (C) The freshness K value of 24 h (*n* = 3); (D) The freshness K value of 48 h (n = 3); (E-H) The adenosine triphosphate (ATP), adenosine monophosphate (AMP), inosinemonophosphate (IMP), inosine (HxR) content of muscle tissue (n = 6); (I-J) The malondialdehyde (MDA) and protein carbonyl (PC) content of muscle tissue (n = 6); (K-L) The catalase (CAT) activity and superoxide dismutase (T-SOD) of muscle tissue (n = 6); (M-N) The pH value of muscle tissue (n = 6); (O) The lactic acid content of muscle tissue (n = 6); (P) The GLUT4 activity of muscle tissue (n = 6); Results were expressed as mean ± SEM, indicating significant differences with different letters (*P* < 0.05).

We further tested the effect of MI levels on the taste of raw and cooked grass carp flesh (Fig. 3 A-B). In raw grass carp flesh, compared with the MI deficiency group, the saltiness of the 292 mg MI/kg group was significantly improved, and the sweetness of the 195 mg MI/kg group was significantly enhanced (*P* < 0.05). In cooked grass carp flesh, the compound flavor CPS and PKS and sweetness were significantly increased at 195 mg MI/kg compared with the MI-deficient group (*P* < 0.05).

After 24 h of fish death, no significant difference in the K value was observed between the MI supplementation groups (*P* > 0.05). At 48 h after fish death, the K value decreased with the increase of dietary MI level, and the K value for the group receiving 487 mg MI/kg was significantly lower compared to the group receiving 35 mg/kg (*P* < 0.05). The content of lactic acid and HxR in fish flesh decreased gradually with the increase of MI level in diet, and the content of lactic acid and HxR in 292 to 487 mg MI/kg group showed a significant decrease compared to the deficiency group, and the ATP, AMP and IMP content and GLUT4 activity showed the opposite trend (*P* < 0.05).

The MDA and PC levels in the 98–487 mg MI/kg group exhibited significantly lower values compared to the deficiency group, while CAT and T-SOD showed an opposite trend (*P* < 0.05). Compared with the MI deficiency group, the fish fed the diet supplemented with 292 mg MI/kg had a significant increase in pH_0h_ and pH_24h_ (*P* < 0.05).

### 3.5 Effect of myo-inositol on elastin deposition in grass carp muscle

**Fig. 4:**
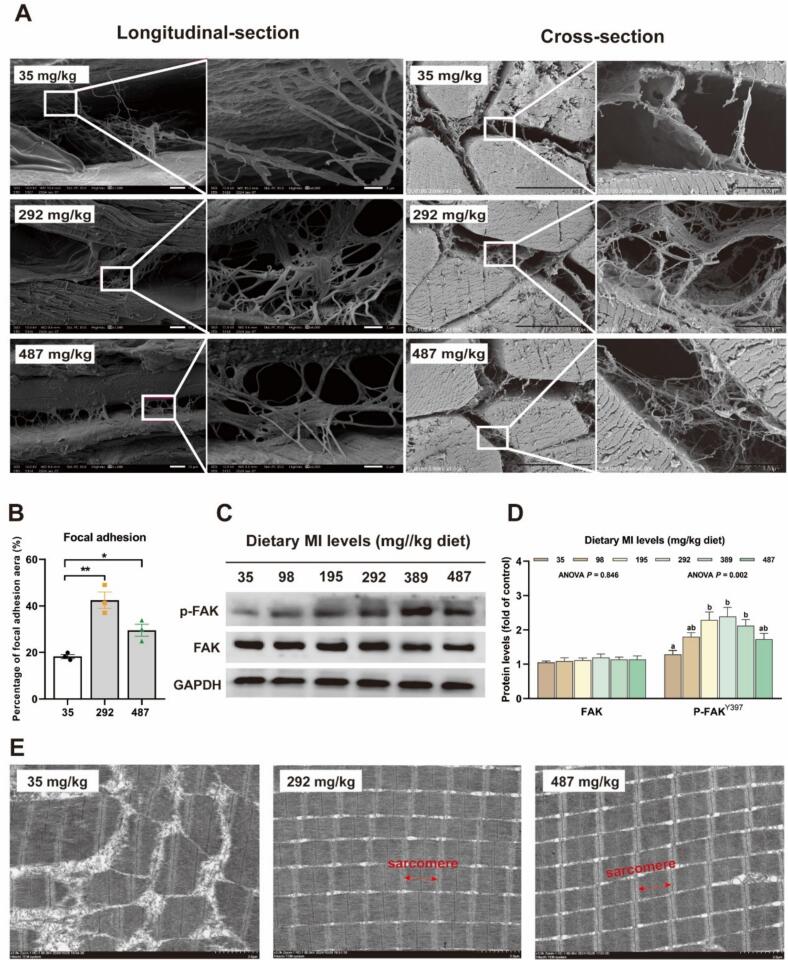

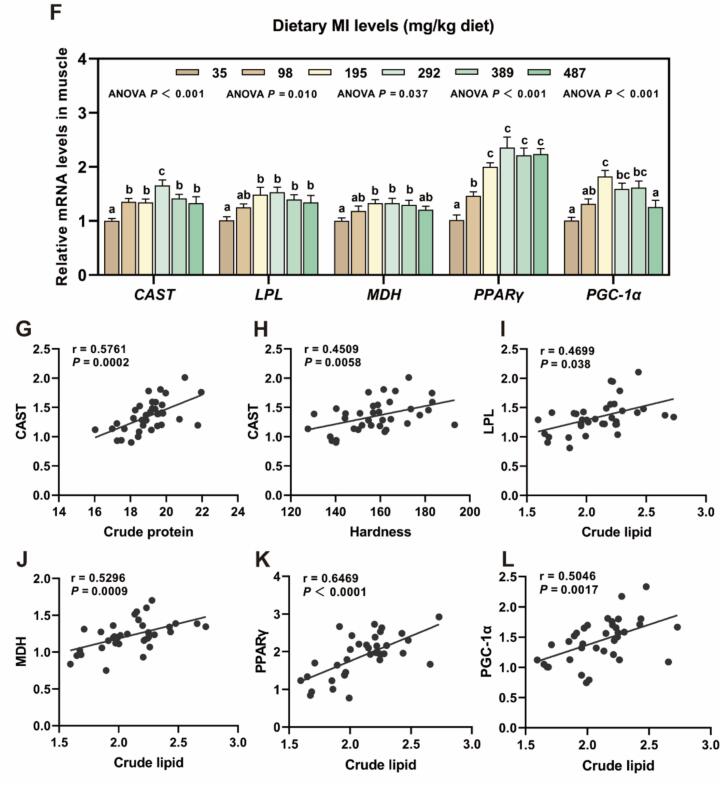
The impact of *myo*-inositol (MI) on muscle nutrition and texture regulation of grass carp. (A-B) The scanning electron microscopy was used to observe the effect of *myo*-inositol (MI) level on muscle focal adhesion area of grass carp (× 1000, left; ×6000, right). Data are presented as mean ± SEM and one-way ANOVA was performed. *P < 0.05, **P < 0.01. (C—D) Protein levels of FAK in grass carp muscle and corresponding quantification analysis (n = 6). (E) The transmission electron microscopy was used to observe sarcomere structure of grass carp muscle (×3000). (F) Key genes involved in muscle nutrition and texture regulation: CAST, LPL, MDH, PPARγ and PGC-1α (n = 6). (G-L) Correlation analysis of muscle nutritional components and texture with (F)figure genes. Results were expressed as mean ± SEM, indicating significant differences with different letters (*P* < 0.05).

As shown in Fig. 4, the SEM analysis revealed a significant increase in the area of focal adhesion for the groups receiving 292 mg MI/kg and 487 mg MI/kg compared to the deficiency group (*P* < 0.05). The protein level of phosphorylated focal adhesion kinase (p-FAK) in the groups supplemented with 195 to 487 mg MI/kg were significantly elevated compared to those in the deficient group (*P* < 0.05).

**Fig. 5:**
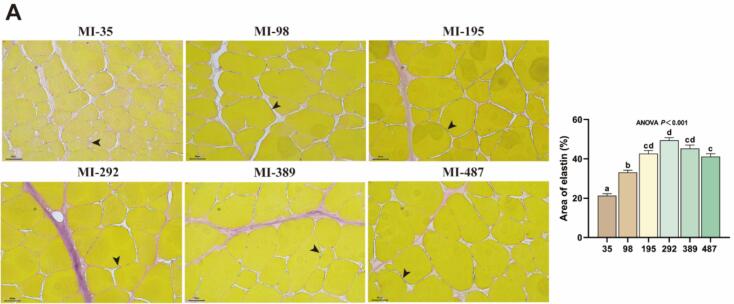

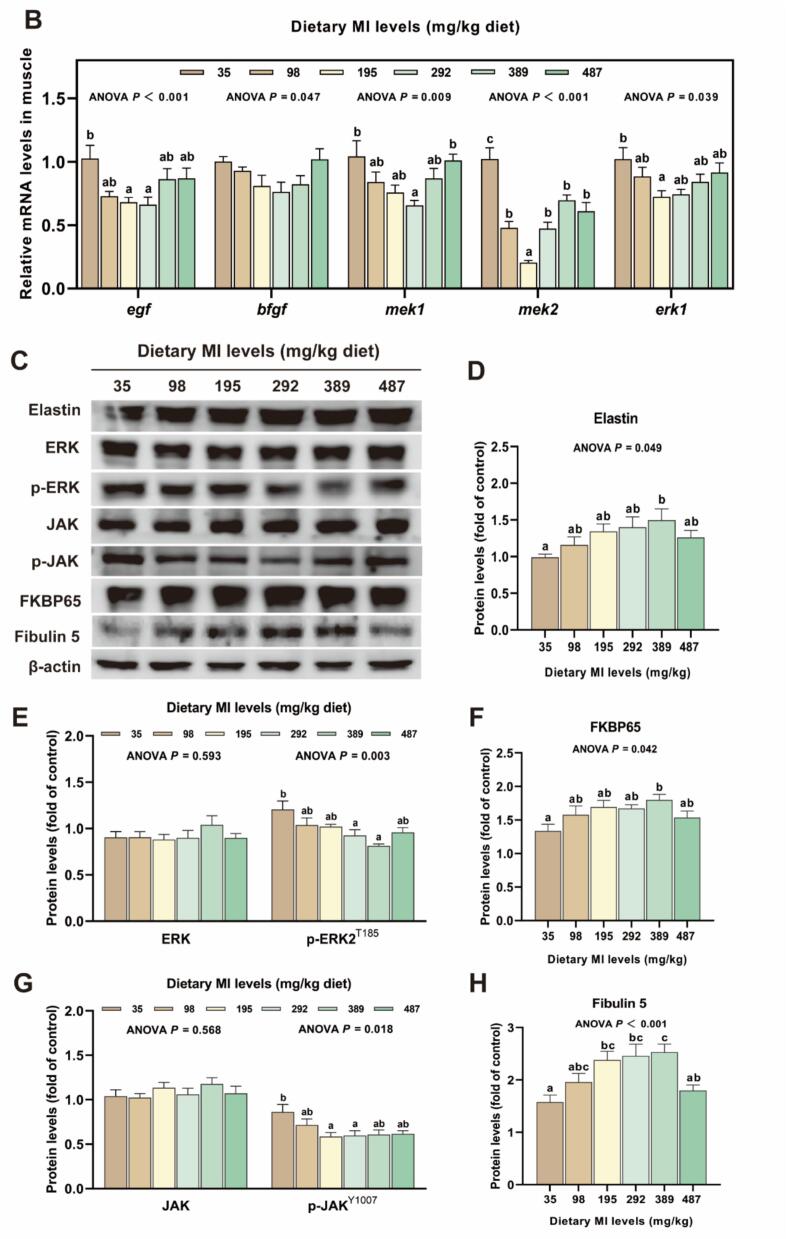
The effects of *myo*-inositol (MI) on elastin synthesis in grass carp muscle. (A) Verhoeff-Van Gieson staining of muscle tissue (magnification 200×; scale bar = 50 μm) (n = 3); (B) Expression of genes associated with elastin synthesis; (C—H) Protein levels associated with elastin transcription and translation in grass carp muscle and corresponding quantification analysis (n = 6). Results were expressed as mean ± SEM, indicating significant differences with different letters (*P* < 0.05).

Elastin synthesis-related proteins are shown in Fig. 5. As shown in Fig. 5A-B, EVG staining of muscle tissue revealed that the area of elastin fibers in the groups receiving 98 to 487 mg MI/kg were significantly higher compared to those in the deficient group (*P* < 0.05). In comparison to the deficient group, the 292 mg MI/kg group exhibited a significant reduction in *egf*, *mek1, mek2* mRNA levels (*P* < 0.05). Compared with the deficiency group, the levels of elastin, 65-kDa FK506-binding protein (FKBP65) and fibulin-5 protein in the 389 mg MI/kg groups were significantly increased, and the levels of p-ERK and phosphorylation-Janus kinase (p-JAK) protein showed the opposite trend (*P* < 0.05). However, no significant differences were observed in the protein levels of ERK and JAK across all groups (*P* > 0.05).

### 3.6 The impact of myo-inositol on the deposition of collagen in grass carp muscle

**Fig. 6:**
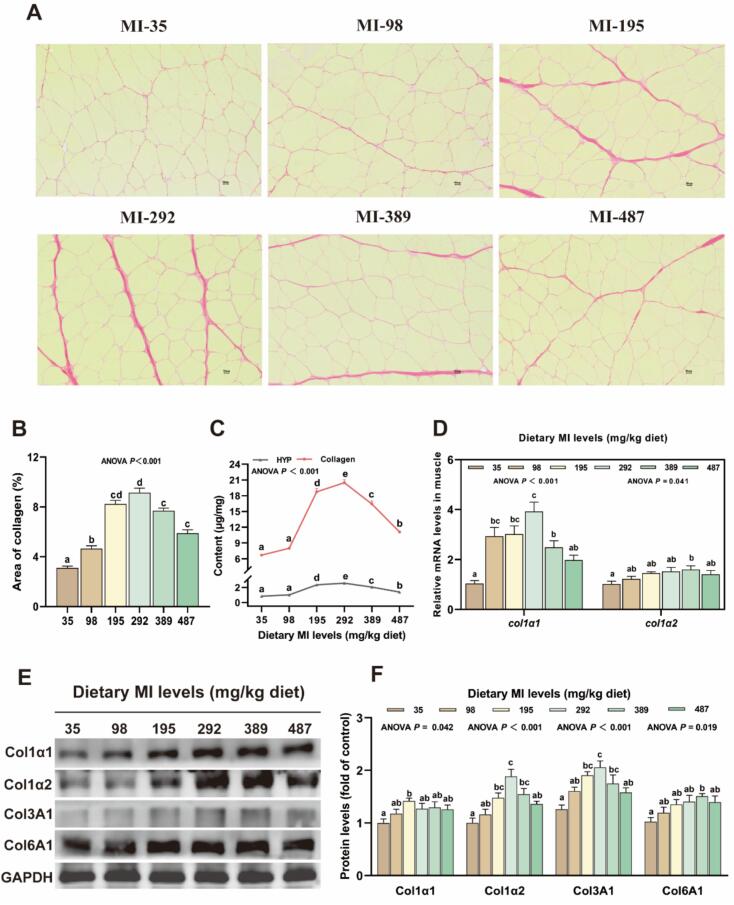
The effects of *myo*-inositol (MI) on collagen content and distribution in grass carp muscle. (A) Sirius red staining of muscle tissue (magnification 200×; scale bar = 50 μm) (n = 3); (B) The proportion of collagen fibers according to statistical analysis of the Sirius red staining (n = 3); (C) The content of HYP and collagen in muscle (n = 6); (D) The mRNA levels of the *col1α1* and *col1α2* in grass carp muscle (n = 6); (E-F) The protein levels of the Col1α1, Col1α2, Col3A1 and Col6A1 in grass carp muscle and corresponding quantification analysis (n = 6). Results were expressed as mean ± SEM, indicating significant differences with different letters (*P* < 0.05). (For interpretation of the references to color in this figure legend, the reader is referred to the web version of this article.)

As shown in Fig. 6A-B, sirius red staining of muscle tissue revealed that the area and content of collagen fibers in the groups receiving 98 to 487 mg MI/kg were significantly higher compared to those in the deficient group (*P* < 0.05). As illustrated in Fig. 6D-F, the mRNA expression of collagen synthesis-related genes *col1α1* and *col1α2*, along with the protein levels of Col1α2, Col3A1, and Col6A1, were markedly elevated in the groups receiving 389 mg MI/kg supplementation compared to the deficient group (*P* < 0.05).

**Fig. 7:**
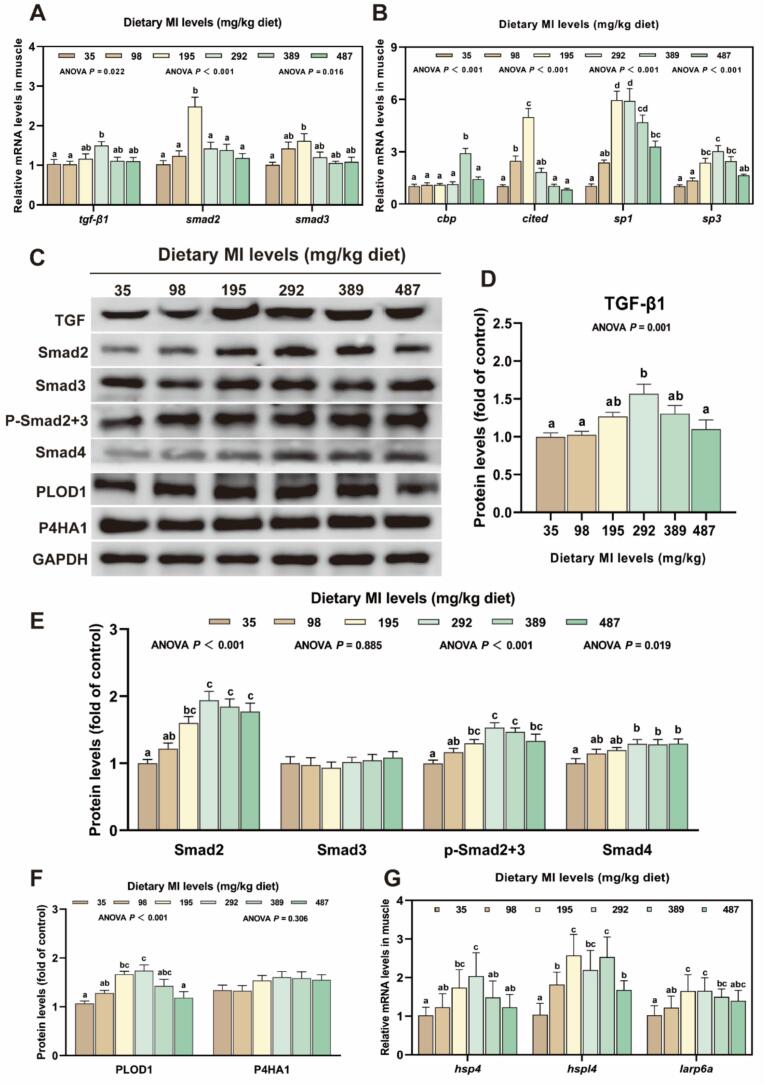

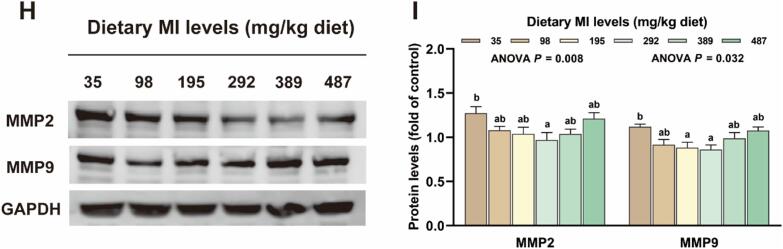
The effect of *myo*-inositol (MI) on the synthesis and degradation of elastin and collagen in grass carp muscle tissue. (A) Expression of genes involved in the transcriptional regulation of elastin and collagen (n = 6); (B) Expression of genes involved in the translational regulation of elastin and collagen (n = 6); (C—F) Protein levels of elastin and collagen transcription and translation in grass carp muscles and corresponding quantification analysis; (G) Expression of genes involved in the post-translational modification of collagen; (H—I) Protein levels of elastin and collagen-degradation in grass carp muscles and corresponding quantification analysis. Results were expressed as mean ± SEM, indicating significant differences with different letters (*P* < 0.05).

In Fig. 7, in comparison to the deficient group, the 195 mg MI/kg group exhibited significantly higher levels of *smad2*, CBP/P300-interacting *trans*-activators with ED-rich tail (*cited*) mRNA as well as Smad2 protein, and the levels of specificity protein 1 (*sp1*), *sp3*, *hsp4*, *hspl4*, *larp6a* mRNA and p-Smad2 + Smad3 and procollagen-lysine, 2-oxoglutarate 5-dioxygenase 1 (PLOD1) proteins were significantly increased in the 195 to 292 mg MI/kg groups (*P* < 0.05). However, no significant differences were observed in prolyl 4-hydroxylase subunit alpha 1 (P4HA1) and Smad3 protein levels between all treatment groups (*P* > 0.05). As shown in Fig. 7 H—I, the protein levels of MMP2 and MMP9 in the 292 mg MI/kg group were significantly decreased compared to the deficient group (*P* < 0.05).

## 4 Discussion

### 4.1 Effects of dietary MI on growth performance of adult grass carp

Our previous research has demonstrated that dietary MI in this experimental formulation can improve the growth performance of adult grass carp. The percentage of weight gain in each group was 54.17 ± 10.20, 84.01 ± 4.12, 86.78 ± 6.33, 89.34 ± 4.80, 88.76 ± 4.48 and 72.73 ± 9.01 %, respectively, as shown in Table S2 (Wang et al., 2025). Adult fish constitutes the principal growth stage selected for human consumption, and the quality of fish holds significant importance. Consequently, we subsequently examined the effects of MI on the texture of adult grass carp flesh.

### 4.2 The impact of myo-inositol on the freshness, antioxidation and flavor of fish flesh

In fish tissues, an autolysis reaction controlled by natural enzymes occurs immediately after death, the main process of which is the decomposition of ATP accompanied by the accumulation of hydrogen ions (Hamm, 1977). The K value, a recognized index of freshness, reflects the rate of ATP catabolism, with elevated K value indicating accelerated ATP degradation [ATP → adenosine monophosphate (AMP) → inosine monophosphate (IMP) → inosine (HxR) → hypoxanthine (Hx)] (Hong et al., 2017). IMP is one of the primary umami nucleotides in fish muscle; its degradation products, HxR and Hx, exhibit a bitter taste. Our findings indicate that MI supplementation did not influence the K value of freshness 24 h post-mortem. After 48 h, our observations revealed a gradual decrease in the K value as the dietary MI level increased. We hypothesize that this phenomenon is likely associated with ATP content in muscle. ATP is predominantly generated through glycolysis in postmortem muscle tissue. Furthermore, previous studies have demonstrated that MI facilitates glucose uptake in rat muscles (Chukwuma et al., 2016). Consequently, we hypothesize that MI enhances ATP content in fish, thereby reducing ATP degradation and subsequently increasing fish freshness. This effect may be linked to the promotion of glucose uptake in muscle tissue. Our findings indicate that MI can enhance the content of ATP, AMP, and IMP in fish muscle while reducing the level of HxR. Meanwhile, the enzymatic activity of GLUT4, the primary glucose transporter protein in skeletal muscle was elevated, thereby providing strong support for our hypothesis.

In general, the acidity of postmortem muscle is attributed to anaerobic glycolysis that converts glycogen into lactic acid. Upon the death of an animal, muscle glycogen is transformed into lactic acid, leading to a reduction in pH levels. In this study, dietary appropriate MI level was found to increase pH_24h_ in muscle tissue while decreasing lactic acid content. This suggested that MI mitigates the decline in pH and reduces the detrimental impact of lactic acid on flesh quality. Based on the above analysis, we hypothesize that MI can enhance the freshness and pH level of fish flesh by leveraging its antioxidant properties, thereby effectively inhibiting the decline in fish flesh quality. The activity of antioxidant enzymes (e.g., SOD and CAT) in muscle tissue plays a critical role in preserving flesh quality post-slaughter. Malondialdehyde (MDA), as a lipid peroxidation product, serves as an indirect indicator of the extent of lipid peroxidation in cells (Dong et al., 2022). Our research findings demonstrate that MI enhances T-SOD and CAT activity in fish muscle, suggesting its potential to sustain muscle antioxidant balance and mitigate oxidative damage. This may partially elucidate how MI contributes to improving fish flesh quality while reducing protein carbonyl (PC) and MDA levels. A recent study on *Procambarus clarkii* has demonstrated that MI can significantly decrease the MDA level in muscle tissue, concurrently enhancing the activities of antioxidant enzymes SOD and CAT, which ultimately leads to an effective improvement in flesh quality (Pu et al., 2025). This finding aligns with the conclusions drawn in this study. Furthermore, the pH value of flesh influences biochemical reactions that generate flavor precursors, thereby affecting the overall flavor and taste of the flesh. Consequently, we further investigated the impact of MI on the flavor characteristics of fish.

Sensory evaluation, as the most direct method for assessing flesh quality, provides valuable insights based on human sensory perception. In this study, we used an electronic tongue device to evaluate the impact of MI on fish flavor. Our findings demonstrated that an appropriate dietary MI enhanced the sweetness of fresh grass carp flesh while reducing its saltiness. In addition, the sweetness and compound flavor of cooked grass carp flesh were improved. At the same time, the umami taste of fresh and cooked flesh both increased at the appropriate MI levels. We speculate that the increase of sweetness and umami in fish may be related to FAA. FAA, as the main water-soluble flavor precursor, plays a crucial role in flavor formation (Wasserman & Gray, 2010). Our findings indicate that appropriate MI levels increased the content of umami free amino acid Glu and sweet free amino acids Thr, Gly and Ala in muscle tissue. These results further confirm that MI can improve the flavor of freshwater fish, but the specific potential mechanism still needs further study.

The formation of muscle nutritional components and texture is a complex biological process that is intricately regulated by multiple genes. Key genes, including CAST, LPL, MDH, PPARγ, and PGC-1α, play pivotal roles in this process. The postmortem softening of fish muscle is commonly perceived as an undesirable quality trait by consumers. This degradation and softening of the muscle are believed to be partially attributed to calpain activity (Gaarder et al., 2011). CAST directly influences muscle tenderness by inhibiting calpain activity and reducing the rate of muscle protein degradation. In this study, we observed that MI enhances the mRNA expression level of CAST. Furthermore, correlation analysis demonstrated that CAST gene expression is positively associated with both the crude protein content in muscle and the hardness parameter of texture properties, suggesting that MI contributes to slowing the degradation of muscle proteins and enhancing flesh quality. LPL and MDH respectively influence muscle lipid accumulation and energy supply via lipid metabolism and the tricarboxylic acid cycle. Previous studies have demonstrated that MI enhances LPL mRNA expression in the livers of juvenile hybrid grouper (*♀ Epinephelus fuscoguttatus x ♂ E. lanceolatu*) and largemouth bass (*Micropterus salmoides*) (Pan et al., 2022; Xu et al., 2024). Our study demonstrated that MI enhances the LPL mRNA expression in the muscle of grass carp. Correlation analysis further indicated that LPL gene expression is positively associated with the crude lipid content in muscle tissue. In hybrid grouper (*Oreochromis niloticus × O. aureus*), the upregulation of LPL gene expression was also positively correlated with muscle lipid content, consistent with the findings of this study (Han et al., 2011). The aforementioned results suggest that MI facilitates lipid accumulation in muscle tissue. A study on the blunt snout bream (*Megalobrama amblycephala*) revealed that reduced MDH activity in muscle tissue adversely affects flesh quality (Bao et al., 2022). Our research revealed that the expression of the MDH gene is positively correlated with intramuscular lipid content in muscle tissue. This may be attributed to dietary MI enhancing mitochondrial biogenesis in muscles, warranting further investigation. PPARγ, a key transcription factor in adipogenesis regulation, collaborates with PGC-1α to modulate mitochondrial biogenesis and fatty acid oxidation, thereby maintaining lipid metabolic balance in muscle tissue. Notably, recent studies have demonstrated that supplementation with MI can enhance PPARγ levels in pig skeletal muscle, leading to increased intramuscular fat (IMF) content and improved pork tenderness (Yan et al., 2024). In this study, the expression levels of PPARγ and PGC-1α genes were positively correlated with intramuscular lipid content in muscle tissue, further substantiating the positive role of MI in lipid deposition.

### 4.3 The effect of myo-inositol on the texture of fish flesh

The texture of flesh is a sensory attribute deemed to be among the most significant factors influencing consumer satisfaction and perception of flesh products. Moreover, texture serves as an indicator of freshness, as flesh spoilage is typically associated with a softening of texture (Szczesniak, 2002). In this study, appropriate MI levels increased muscle hardness, adhesive force, springiness, resilience and chewiness, indicating that MI had the effect of improving flesh texture. We hypothesize that the observed increase in hardness and springiness might be linked to focal adhesions (FA). FA is a macromolecular structure that connects the actin cytoskeleton to the extracellular matrix (ECM). FA has many functions, such as cytoskeleton regulation, force transmission and signal transduction. Our SEM results also confirm that MI can increase the FA area. It is reported that FAK is a key signal transduction regulator of FA. In our study, the appropriate level of MI increased p-FAK protein level. In addition, FAK interacts with the FA membrane by binding to the MI derivative phosphatidylinositol 4,5-diphosphate (PI (4, 5) P_2_) (Le Coq et al., 2022). Thus, these findings suggest that an appropriate dietary MI may elevate FAK expression, influencing the FA area and consequently improving flesh quality. However, the precise mechanism requires further investigation.

The sarcomere serves as the fundamental structural and functional unit of muscle tissue. It maintains the structural integrity of the muscle while facilitating signal transmission. Additionally, the contractile force generated by sarcomeres determines the elastic properties of striated muscles (Tskhovrebova & Trinick, 2010). In our prior laboratory research, the deficiency of MI was found to result in skin and muscle ulceration in Jian carp (unpublished data). In this study, transmission electron microscopy (TEM) observations revealed that MI deficiency leads to disorganization of the sarcomere structure, suggesting that MI deficiency may compromise the structural integrity and elastic properties of muscle tissue. This phenomenon is likely attributable to inositol serving as a precursor for inositol triphosphate (IP_3_), with IP_3_ receptor (IP_3_R) functioning as key channel of intracellular Ca^2+^ release, thereby modulating Ca^2+^ signaling. It has been reported that Ca^2+^ signals occur both in vivo and in vitro, and blocking their generation can inhibit the assembly of sarcomeres (Ferrari et al., 2006). Furthermore, MI was initially discovered in muscle tissue and is known as muscle ‘sugar’ (Shirmohammad et al., 2016). Therefore, the deficiency of MI results in disorganization of the muscle sarcomere structure, which in turn compromises the overall structure and function of the muscle.

Alterations in flesh texture are influenced by numerous factors, such as diet, myofiber diameter, and intramuscular connective tissue (Wang et al., 2024). Our previous studies have shown that MI promoted the increase of myofiber diameter (Wang et al., 2025). Notably, several studies have suggested a correlation between increased muscle hardness and reduced myofiber size. Our experimental results may conflict. This outcome may be attributed to the interplay of multiple factors that collectively determine the overall textural properties of flesh, rather than solely the physical structure of the myofibers.

### 4.4 Dietary myo-inositol regulates the biosynthesis of elastin in grass carp flesh

Elastin constitutes theprimary component of elastic fibers and serves as a crucial structural element of the ECM, significantly contributing to its mechanical integrity and elasticity (Yeo et al., 2021). In this investigation, the appropriate MI levels was found to elevate elastin protein levels. The expression and formation of elastin are regulated by a variety of effector factors. Among these, TGF-β1/Smads promotes elastin synthesis by enhancing proelastin promoter activity and mRNA stability (Sproul & Argraves, 2013). Our findings indicate that optimal levels of MI increase the expression of TGF-β1, as well as the protein levels of p-Smad2 + 3 and Smad4 in muscle. These findings indicate that MI stimulates collagen transcription through the activation of the TGF-β1/Smads signaling cascade. Contrary to TGF-β1 signal, the EGF, bFGF/MEK/ERK signaling cascade mitigates the stabilizing effect of TGF-β1 on elastin mRNA (DiCamillo et al., 2006). In this study, MI reduced the mRNA levels of *egf*, *mek1*, *mek2*, and *erk1*, and the protein level of p-ERK. Additionally, JAK signal transduction is pivotal in regulating cytokine and certain growth factor-induced signaling pathways, thereby affecting the process of inflammation and fibrosis (Raychaudhuri et al., 2021). In a study investigating mouse vascular smooth muscle, it was demonstrated that inhibition of the JAK pathway facilitated the restoration of elastin within the mouse vascular wall (Jiang et al., 2021). This study further demonstrated that appropriate MI levels leads to a reduction in p-JAK protein levels. FKBP65, an endoplasmic reticulum-resident peptidyl-prolyl isomerase, functions as an intracellular chaperone for proelastin and serves a critical function in ensuring the correct folding of proelastin molecules (Davis, 1998). The findings of this study indicate that appropriate MI levels increases the protein level of FKBP65 in muscle, suggesting that MI enhances the stability of proelastin molecules. Fibulin-5 is an extracellular matrix glycoprotein expressed in elastin-rich tissues, playing a critical role in regulating elastin fiber deposition. Research has demonstrated that mice deficient in fibulin-5 exhibit widespread loosening of elastin fibers (Nakamura et al., 2002). Moreover, fibulin-5 interacts with cross-linking enzymes, such as lysyl oxidase (LOX). Fibulin-5 facilitates the transport of secreted proelastin to the microfibril assembly site, where LOX interacts with fibulin-5 to facilitate the crosslinking of proelastin monomers into insoluble elastin polymers (Sato et al., 2007). This study showed that an appropriate dietary intake of MI also elevated the protein levels of fibulin-5. Furthermore, our previous studies demonstrated that the MI elevated the protein expression of LOX in grass carp muscle. These results suggest that MI enhances elastin deposition, potentially through fibulin-5-mediated promotion of elastin cross-linking. Consequently, we conjecture that MI may modulate the expression of fibulin-5 via promoting TGF-β1/Smads, inhibiting EGF, bFGF/MEK/ERK, and JAK pathways, thereby enhancing elastin biosynthesis and increasing the springiness of flesh.

### 4.5 Dietary myo-inositol regulates the biosynthesis of collagen in grass carp flesh

Collagen, as the primary constituent of connective tissue, is intricately associated with muscle hardness. This investigation demonstrated that supplemented with MI exhibited an elevated collagen content within the muscle. In *procambarus clarkii*, it was also observed that dietary MI can enhance the collagen content in muscle tissue, which is in agreement with the findings of this study. Additionally, sirius red staining revealed an expansion in the area of collagen fibers, suggesting that dietary MI promoted collagen synthesis, thereby improving flesh quality. Given that collagen biosynthesis is influenced by multiple factors, we further investigated the underlying mechanisms by which MI affects collagen biosynthesis.

Collagen encompasses a diverse array of types with distinct compositions. Notably, type I (Col1α1 and Col1α2) and type III (Col3A1) collagen constitute the predominant forms within muscle tissue. Type I collagen is distinguished by its ability to form robust, parallel fibers, thereby imparting tensile strength and stiffness. In contrast, type III collagen generates a more flexible fiber network, contributing to tissue compliance (Kovanen, 2002). Additionally, type VI collagen (Col6A1), synthesized by endomysial cells, serves as a crucial survival factor for skeletal muscle and represents the primary collagen component of all basement membranes throughout the body (Braghetta et al., 2008). This study demonstrated that an appropriate dietary intake of MI elevated the mRNA levels of *col1α1* and *col1α2*, and the protein levels of COL1A1, COL1A2, COL3A1, and COL6A1. These results suggest that MI enhances collagen biosynthesis. This phenomenon may be linked to the TGF-β1 signaling pathway. The TGF-β1/Smads is widely acknowledged as the primary mechanism governing the transcription of collagen genes. The Smad family, functioning as signal transduction proteins, facilitates TGF signal transduction from the cell surface to the nucleus through interactions with transcriptional coactivators such as CREB binding protein (CBP), CITED, and SP1/3 (Ismaeel et al., 2019). In the present study, the appropriate MI levels resulted in elevated mRNA expression levels of *tgf-β1*, *smad2*, *smad3*, *sp1*, and *sp3*, as well as increased protein levels of TGF-β1, Smad2, p Smad2 + Smad3, and Smad4. Consequently, MI may enhance collagen transcription through the activation of the TGF-β1/Smads and CITED signaling pathways. The aforementioned findings indicate that MI could facilitate collagen synthesis may be associated with enhanced collagen transcription.

During the translation phase of collagen synthesis, the transcription of procollagen is modulated by LARP6a, while and the effective formation and folding of collagen-specific molecular chaperone Hsp47/Hsp47-like modified procollagen chains (Liang et al., 2021). Additionally, PLOD1 and PHA1 play crucial roles in the hydroxylation of intracellular procollagen chains, contributing to the maturation of collagen fibers (Gelse et al., 2003). This research demonstrated that an appropriate dietary intake of MI elevated *larp6a*, *hsp4* and *hspl4* mRNA expression, and the protein levels of PLOD1. These findings suggest that MI enhances collagen translation and post-translational modification in muscle. Furthermore, previous studies have shown that HSP47 interacts with FRBP65 to enhance the molecular stabilization and post-translational modification of type I procollagen (Ito & Nagata, 2019). Concurrently, appropriate levels of MI in this study were associated with increased FKBP65 protein levels in muscle, suggesting that MI facilitates proper folding of the procollagen molecule. This process of promoting collagen translation and post-translational modification may be associated with IGF-1. Previous studies have reported that IGF-1 induces *larp6a* mRNA expression in human smooth muscle cells to increase collagen synthesis (Blackstock et al., 2014). On the one hand, MI has insulin-like activity, on the other hand, our study also found that MI can up-regulate the level of *igf-1* mRNA in grass carp muscle (Wang et al., 2025). Based on the above results, appropriate dietary MI to increase the expression of LARP6a may be related to IGF-1, thereby promoting the translation and post-translational modification of collagen in grass carp muscle. Nevertheless, additional studies are needed to elucidate the exact mechanism in fish.

### 4.6 The inhibitory effect of myo-inositol on the degradation of elastin and collagen was related to MMP

MMPs form a family of zinc-containing, calcium-dependent endopeptidases that are essential for tissue remodeling and the degradation of ECM components such as elastin and collagen. Among MMPs, gelatinases (MMP-2 and MMP-9) are the most extensively studied enzymes that can effectively cleave the triple helical region of elastin and collagen molecules (Wu et al., 2014). The findings of this research showed that appropriate MI levels reduced the protein levels of MMP-2 and MMP-9, suggesting that appropriate MI levels may slow elastin and collagen degradation. However, the precise mechanism by which MI modulates MMP activity to retard elastin degradation remains to be elucidated, warranting further investigation.

### 4.7 High levels of myo-inositol adversely influenced grass carp flesh quality

As a nutrient with vitamin-like properties, MI has not been associated with any significant adverse effects on the growth performance or health status of juvenile grass carp to date, according to the findings of this study (Li et al., 2017b). However, the present study revealed that under high MI supplementation levels (487 mg/kg), the elastin and collagen content in grass carp muscle was reduced compared to the optimal MI supplementation range. Additionally, high MI levels were associated with an upregulation of MMP2 and MMP9 protein expression. These findings indicate that excessive MI supplementation may negatively impact the muscle quality of grass carp. We hypothesize that the possible reason for the non-linear change in collagen content and elastin is low-dose MI promotes collagen and elastin synthesis by activating the PI3K/AKT signaling pathway, as evidenced by the upregulation of COL1A1 and elastin gene expression. However, under high-dose conditions, it may trigger a negative feedback mechanism, such as inhibiting inositol phosphate synthase (IPS) activity, which impedes phosphatidylinositol (PI) production and ultimately attenuates the transmission of collagen and elastin synthesis-related signals.

## 5 Conclusions

The study examined the impact of dietary MI on the springiness and hardness of fish flesh texture. According to the quadratic regression curves of collagen content, MI requirements for adult grass carp was 269.58 mg/kg diet (Table S5). Sensory evaluation and texture analysis demonstrated that MI facilitates the synthesis of elastin and collagen in the muscle of grass carp while inhibiting their degradation, thus enhancing the springiness and hardness of the fish flesh. Furthermore, texture is an indicator of freshness, as postmortem fish typically exhibit rapid softening. Our findings indicate that dietary MI can effectively slow the loss of freshness in fish post-slaughter and enhance flesh flavor. These results offer a theoretical foundation and elucidate the molecular mechanisms by which dietary MI contributes to the improvement of flesh quality. In future studies, we will further investigate and validate the regulatory effects of MI on the upstream and downstream mechanisms of MMP in vivo.

## CRediT authorship contribution statement

**Meiqi Wang:** Writing – original draft, Visualization, Validation, Software, Investigation, Conceptualization. **Lin Feng:** Methodology, Formal analysis, Data curation. **Pei Wu:** Methodology. **Yang Liu:** Methodology. **Hongmei Ren:** Software, Project administration, Methodology. **Xiaowan Jin:** Visualization, Project administration. **Xiaoqiu Zhou:** Validation, Supervision, Resources, Funding acquisition. **Weidan Jiang:** Writing – review & editing, Validation, Supervision, Methodology, Funding acquisition.

## Ethical statement

All procedures in the animal experiment were conducted in accordance with the Guidelines for the Care and Use of Laboratory Animals in China. This study received approval from the Animal Care Advisory Committee of Sichuan Agricultural University (No. WMQ-2022114015, Date: 09/2022–06/2026).

## Declaration of competing interest

The authors declare that they have no known competing financial interests or personal relationships that could have appeared to influence the work reported in this paper.

## Data Availability

All data generated or analyzed during this study are included in this published article and its supplementary information files.

## Appendix A Supplementary data

Supplementary material Image 1
