# Supplementary material for: Regulation of muscle springiness and hardness: the role of myo-inositol in enhancing fish flesh texture
Source: Food Chem X. 2025 Jul 12;29:102788. doi: 10.1016/j.fochx.2025.102788 (PMC12284545; doi:10.1016/j.fochx.2025.102788)
Supplement: Supplementary file 1 — Supplementary material [file mmc1.docx]

**Supplementary materials**

**Regulation of muscle springiness and hardness: the role of *myo*-inositol in enhancing fish flesh texture**

Meiqi Wang ^a^, Lin Feng ^a,b,c^, Pei Wu ^a,b,c^, Yang Liu ^a,b,c^, Hongmei Ren ^a,b,c^, Xiaowan Jin ^a,b,c^, Xiaoqiu Zhou ^a,b,c*^, Weidan Jiang ^a,b,c*^

^a^ Animal Nutrition Institute, Sichuan Agricultural University, Chengdu, 611130, China

^b^ Fish Nutrition and Safety Production University Key Laboratory of Sichuan Province, Sichuan Agricultural University, Chengdu, 611130, China

^c^ Key Laboratory of Animal Disease-Resistance Nutrition, Ministry of Education, Ministry of Agriculture and Rural Affairs, Key Laboratory of Sichuan Province, Sichuan, 611130, China

^*^ Corresponding author: Animal Nutrition Institute, Sichuan Agricultural University, Chengdu,611130, Sichuan, China

E-mail addresses: zhouxq@sicau.edu.cn (Xiaoqiu Zhou), WDJiang@sicau.edu.cn (Weidan Jiang).

**Table S1**

**Ingredients and nutrient composition of the diet (air-dry basis, g/kg).**

| Ingredients | Content | Nutrient levels^5^ | Content |
| --- | --- | --- | --- |
| Fish meal (CP, 66.25%) | 100.00 | Crude protein | 267.24 |
| Casein (CP, 75.87%) | 170.00 | Crude lipid | 41.81 |
| Gelatin (CP, 82.68%) | 73.98 | ω-3 PUFA | 9.95 |
| Fish oil | 20.20 | ω-6 PUFA | 9.65 |
| Soybean oil | 18.20 | Available phosphorus | 4.00 |
| Corn starch | 188.38 | Organic matter | 853.50 |
| a-Starch | 300.00 | Gross energy, MJ/kg | 17.57 |
| Microcrystalline cellulose | 65.00 |  |  |
| CaH_2_PO_4_ | 11.30 |  |  |
| Mineral premix^1^ | 20.00 |  |  |
| Vitamin premix (myo-inositol free)^2^ | 10.00 |  |  |
| Choline chloride^3^ | 10.00 |  |  |
| Myo-inositol premix^4^ | 10.00 |  |  |
| Butylated hydroxyanisole | 0.15 |  |  |
| L-Thr（97.5%） | 0.91 |  |  |
| L-Trp（98%） | 1.88 |  |  |
| Total | 1000.00 |  |  |

CP = crude protein; PUFA = polyunsaturated fatty acids.

^1^ Per kilogram of mineral premix (g/kg): MnSO_4_•H_2_O (31.8% Mn), 2.66; MgSO_4_•H_2_O (15.0% Mg), 256.79; FeSO_4_•H_2_O (30.0% Fe), 12.61; ZnSO_4_•H_2_O (34.5% Zn), 8.87; CuSO_4_•5H_2_O (25.0% Cu), 0.95; Ca (IO_3_)_2_ (3.2% I), 1.56; selenium yeast (0.2% Se) 13.65. All ingredients were diluted with corn starch to 1 kg.

^2^ Per kilogram of vitamin premix (g/kg):VA (500,000 IU/g), 0.44; VD_3_ (500,000 IU/g), 0.19; VE (50%), 25.50; VK_3_ (50%), 0.38; VB_12_ (1%), 0.94; D-biotin (2%), 1.05; folic acid (95%), 0.17; thiamin nitrate (98%), 0.11; VC (95%), 9.77; nicotinic acid (99%), 3.44; calcium pantothenate (93.1%), 4.42; riboflavin (80%), 0.73; VB_6_ (98%), 0.55. All ingredients were diluted with corn starch to 1 kg.

^3^ Per kilogram of choline chloride, which contains choline chloride (50%) 306.71 g, the rest is diluted with corn starch to 1 kg.

^4^ Provided the following per kilogram of myo-inositol (MI) premix: from treatments 1 to 6, the supplementation of MI at 0.000, 0.067, 0.171, 0.274, 0.378, 0.481 g, respectively, and filled with microcrystalline cellulose to 1 kg.

^5^ Available phosphorus, ω-3 PUFA, and ω-6 PUFA were calculated by NRC (2011).

**Table S2 Effect of dietary *myo*-inositol on growth performance of adult grass carp (*Ctenopharyngodon idella*).**

| Item | Dietary MI levels, mg/kg diet | | | | | | SEM | *P*-value | | |
| --- | --- | --- | --- | --- | --- | --- | --- | --- | --- | --- |
|  | 35.38 | 98.12 | 195.21 | 292.30 | 389.39 | 486.48 |  | ANOVA | Linear | Quadratic |
| IBW^1^, g/fish | 705.80 | 703.50 | 705.10 | 704.00 | 705.00 | 705.62 | 0.391 | 0.314 | 0.811 | 0.249 |
| FBW^1^, g/fish | 1008.03^a^ | 1295.47^bc^ | 1318.68^c^ | 1331.69^c^ | 1330.13^c^ | 1218.53^b^ | 23.985 | ＜0.001 | 0.007 | ＜0.001 |
| PWG^1^, % | 54.17^a^ | 84.01^bc^ | 86.78^c^ | 89.34^c^ | 88.76^c^ | 72.73^b^ | 3.415 | ＜0.001 | 0.006 | ＜0.001 |
| SGR^1^, %/d | 0.77^a^ | 1.09^bc^ | 1.11^c^ | 1.14^c^ | 1.13^c^ | 0.97^b^ | 0.036 | ＜0.001 | 0.005 | ＜0.001 |
| FI^1^, g/fish | 1065.76^a^ | 1215.97^bc^ | 1220.27^c^ | 1220.69^c^ | 1221.17^c^ | 1205.98^b^ | 13.759 | ＜0.001 | ＜0.001 | ＜0.001 |
| FE^1^ | 0.36^a^ | 0.49^bc^ | 0.50^bc^ | 0.51^c^ | 0.51^c^ | 0.43^ab^ | 0.016 | 0.003 | 0.057 | ＜0.001 |
| Whole length^2^, cm | 43.42^a^ | 45.67^b^ | 48.25^d^ | 47.50^cd^ | 47.58^cd^ | 46.50^bc^ | 0.319 | ＜0.001 | ＜0.001 | ＜0.001 |
| Body length^2^, cm | 37.00^a^ | 39.17^b^ | 41.50^d^ | 40.75^cd^ | 40.75^cd^ | 39.67^bc^ | 0.290 | ＜0.001 | ＜0.001 | ＜0.001 |
| Body width^2^, cm | 5.15^a^ | 5.42^b^ | 5.50^bc^ | 5.75^c^ | 5.73^c^ | 5.58^bc^ | 0.049 | ＜0.001 | ＜0.001 | ＜0.006 |
| Body height^2^, cm | 8.00^a^ | 8.70^b^ | 9.13^c^ | 9.25^c^ | 9.28^c^ | 9.05^c^ | 0.086 | ＜0.001 | ＜0.001 | ＜0.006 |
| CF^2^, g/cm^3^ | 1.84^a^ | 1.88^a^ | 1.99^b^ | 2.00^b^ | 2.02^b^ | 1.92^ab^ | 0.017 | 0.006 | 0.012 | 0.003 |
| VSI^2^, % | 10.43 | 10.65 | 11.33 | 11.20 | 11.07 | 11.04 | 0.193 | 0.786 | 0.325 | 0.327 |
| HSI^2^, % | 2.79 | 2.81 | 2.90 | 3.12 | 2.94 | 2.91 | 0.045 | 0.334 | 0.192 | 0.192 |
| **Muscle composition, %** | | | | | | |  |  |  |  |
| Moisture^2^ | 79.15^b^ | 78.36^ab^ | 77.88^a^ | 77.46^a^ | 77.67^a^ | 78.05^a^ | 0.153 | 0.012 | 0.006 | 0.006 |
| Crude protein^2^ | 17.37^a^ | 18.67^b^ | 19.63^bc^ | 20.12^c^ | 19.71^bc^ | 18.67^b^ | 0.209 | ＜0.001 | 0.003 | ＜0.001 |
| Crude lipid^2^ | 1.74^a^ | 1.89^a^ | 2.24^bc^ | 2.41^c^ | 2.18^b^ | 2.14^b^ | 0.045 | ＜0.001 | ＜0.001 | ＜0.001 |

MI = myo-inositol; IBW = initial body weight; FBW = final body weight; FI = feed intake; FE = feed efficiency; PWG = percent weight gain; SGR = specific growth rate; CF = condition factor; VSI = viscerosomatic index; HSI = hepatosomatic index.

^a-d^ Mean values within a row with different superscript letters indicate significant difference (*P* < 0.05).

^1^ *n* = 3 (for 3 replicate groups, 25 fish per replicate).

^2^ *n* = 3 (for 3 replicate groups, 2 fish per replicate).

Mean values within the same row with different superscripts are significantly different (*P* < 0.05), this supplementary material is according to our previous study (Wang et al., 2025).

**Table S3. The primer sequences and accession numbers of genes selected for analysis by real-time PCR.**

| Target gene | Primer sequence forward (5′ → 3′) | Primer sequence reverse (5′ → 3′) | Accession number |
| --- | --- | --- | --- |
| *tgf-β1* | TTGGGACTTGTGCTCTAT | AGTTCTGCTGGGATGTTT | EU099588 |
| *smad2* | GTCCTCCATCTTGCCTTTCAC | CTTCTCGCACCATTTCTCCTC | DQ912858 |
| *smad3* | ATTGAGCCTCCGAGCAACTAT | GAAAGATTTGGGGAACCTGTG | DQ912859 |
| *larp6a* | CTGAGGAGTGTGCCATCGTAG | TTCTTGGGAGGTTTGGTGCC | OL438919 |
| *col1α1* | CAACAGCCGCTTCACATACA | GGCGATGTCAATAATAGGCAG | HM363526 |
| *col1α2* | CAAGAACAGCATCGCCTACAT | AGATGGTTTATTCGTTCTGTATTCA | HM771241 |
| *sp1* | TTCTGCGGGAAACGTTTCAC | ACTACGCATGAACCGTTTGG | KY081668 |
| *sp3* | AGCACCTCATGACCCAGTCT | TTCTCCGAGCCTTCAGAGTT | KJ095608 |
| *hsp47* | TGAGCTGGGTCTGACCGAAG | TGAAGGGATGGTCAGCGTAG | MT657345 |
| *hsp47like* | CGTGGACAAGGCTAAGGCAGAC | GCATGGAAGACATTGGCGAGGTAA | MW263909 |
| *crebp* | GGCTGAGAGAGTTCGGCAAAC | CATTTGGCAGTGATCCTCCAT | EF437961 |
| *cited1* | TGATGCCTATGAACCATGGT | CAGTACTTGGCTGCTGCTTG | EU450669 |
| *mek1* | ACATAGTGGGCTTCTACGGG | TGCCTGCTTTCTTCAGGGAC |  |
| *mek2* | GCACGCACTATTCGGTTCAG | CAGTTCCTTGGCATCGGGAG |  |
| *erk1* | CCTGCGAGGGCTGAAATACA | TGTGGTCATGTTCTGGGTCG |  |
| *egf* | CTGGTGTGACCTGAGCAGAG | ACGTCAAACGGGTGACCTAC | XM051861038.1 |
| *bfgf* | TTCGGGGAACTTCAAGGAGC | CGTCCGAGTTGATCCGTAGG | XM051860605.1 |
| *CAST* | CACACACGCTTTGATTCTCCC | CACACACGCTTTGATTCTCCC | XM_051877319.1 |
| *LPL* | GTTCGCTCATTCAGGGTGGA | GTTCGCTCATTCAGGGTGGA | XM_051878965.1 |
| *MDH* | ACACTCCGCACGAAAGTAGG | TAGCCGACAACAGGGTCAAC | XM_051910623.1 |
| *PPARγ* | CGGATATGGTGGACACGCAG | GCTGTTCTCGGCAAACTGTG | XM_051913344.1 |
| *PGC-1α* | TACTCAAGGGGAGCAATCCGC | TCATGCAAGCTGTCTCCTGT | XM_051900017.1 |
| *β-actin* | GGCTGTGCTGTCCCTGTA | GGGCATAACCCTCGTAGAT | M25013 |

**Table S4. Primary antibodies for the Western blot analyses.**

| Target proteins | Dilution factor | Antibody cat. no. | Antibody source |
| --- | --- | --- | --- |
| FAK | 1:1000 | A1131 | ABclonal, China |
| p-FAK | 1:1000 | AP1447 |  |
| PLOD1 | 1:1000 | A7979 |  |
| P4HA1 | 1:1000 | A10538 |  |
| Col1α1 | 1:1000 | A1352 |  |
| Col1α2 | 1:1000 | A5786 |  |
| Col3A1 | 1:1000 | A0817 |  |
| Col6A1 | 1:1000 | A9236 |  |
| TGF-β1 | 1:1000 | A2124 |  |
| Smad2 | 1:1000 | A0440 |  |
| Smad3 | 1:1000 | A16913 |  |
| Smad4 | 1:1000 | A5657 |  |
| p-Smad2+Smad3 | 1:1000 | AP1343 |  |
| MMP2 | 1:1000 | A6247 |  |
| MMP9 | 1:1000 | A25299 |  |
| Elastin | 1:1000 | ER1908-02 | Huaan Biotechnology |
| Fibulin 5 | 1:1500 | ER1802-95 |  |
| ERK | 1:1000 | ET1601-29 |  |
| P-ERK | 1:1000 | ET1610-13 |  |
| JAK | 1:1000 | ET1607-35 |  |
| p-JAK | 1:1000 | ET1607-34 |  |
| FKBP65 | 1:5000 | 12172-1-AP | proteintech |

**Table S5. The quadratic regression curve of collagen content.**


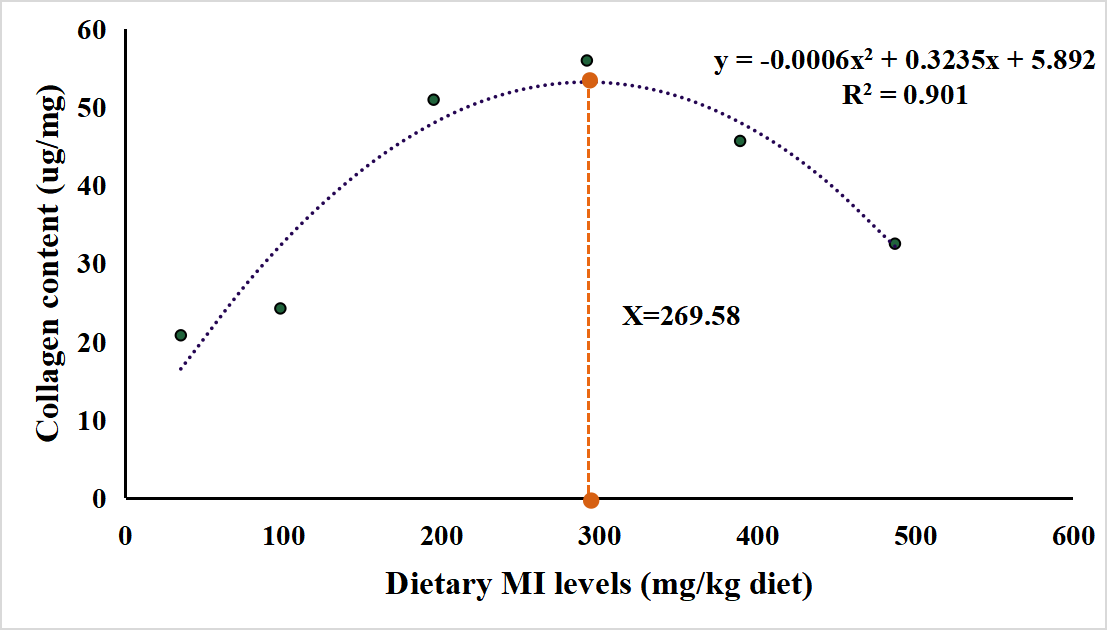


**Reference**

Wang, M. Q., Feng, L., W, P., Liu, Y., Ren, H. M., Jin, X. W., Zhou, X. Q., & Jiang, W. D. (2025). Multiple ways to promote adult grass carp (*Ctenopharyngodon idella*) muscle hypertrophy: Application of dietary *myo*-inositol. *Animal Nutrition*.<http://dx.doi.org/https://doi.org/10.1016/j.aninu.2025.01.010>
